# GC‐MS Analyses and Bioactivity Evaluation of *Origanum minutiflorum* Essential Oil: Antioxidant, Antibacterial, Genotoxicity, and Molecular Docking Studies

**DOI:** 10.1002/cbdv.71725

**Published:** 2026-09-22

**Authors:** Derya Efe, Ayça Aktaş Karaçelik, Sedat Bozarı, Gözde Yalçın Özkat, Büşra Korkmaz

**Affiliations:** ^1^ Espiye Vocational School, Department of Medicinal and Aromatic Plants Giresun University Giresun Türkiye; ^2^ Espiye Vocational School, Department of Food Processing Giresun University Giresun Türkiye; ^3^ Faculty of Arts and Sciences, Department of Molecular Biology and Genetics Muş Alparslan University Muş Türkiye; ^4^ Bioengineering Department Faculty of Engineering and Architecture Recep Tayyip Erdogan University Rize Türkiye; ^5^ Department of Pharmacognosy Faculty of Pharmacy Karadeniz Technical University Trabzon Türkiye

**Keywords:** ADME, biological activity, GC‐MS, genotoxic, molecular docking, molecular dynamics, *Origanum minutiflorum*, RAPD

## Abstract

This study aimed to elucidate the chemical composition of *Origanum minutiflorum* essential oil (OmEO) and evaluate its antioxidant and antibacterial activities, while also investigating, for the first time, the antibacterial potential of its major volatile constituents using in silico approaches, including ADME profiling and molecular docking, together with a genotoxicity assessment. Thirteen constituents, accounting for 99.10% of the total oil composition, were identified, with *p*‐cymene (36.7%), *γ*‐clausenane (31.1%), and borneol (10.6%) as the major components. OmEO exhibited moderate antioxidant activity, with IC_50_ values of 1.0864 ± 0.0296 mg/mL in the DPPH• assay and 0.0055 ± 0.0002 mg/mL in the ABTS•^+^ assay, as well as a TEAC value of 745.56 ± 1.47 µM. The total phenolic content was 558.57 ± 1.43 µg/mL GAE. OmEO exhibited dose‐dependent antibacterial activity against both Gram‐positive and Gram‐negative bacteria, with a MIC of 50 µg/mL for all tested strains except *Enterobacter aerogenes*, for which the MIC was 100 µg/mL. In the genotoxicity assessment, the oil inhibited stem elongation more strongly than root growth. In silico analyses identified borneol and *γ*‐clausenane as promising candidates for further investigation of their antibacterial potential.

## Graphical abstract

GC–MS analysis reveals the chemical composition of *Origanum minutiflorum* essential oil, while biological and computational investigations demonstrate its antioxidant, antibacterial, and genotoxic properties. Favorable ADME profiles and molecular interactions of the major terpenoids highlight their potential as natural antibacterial agents and promising bioactive compounds for future pharmaceutical and food applications.

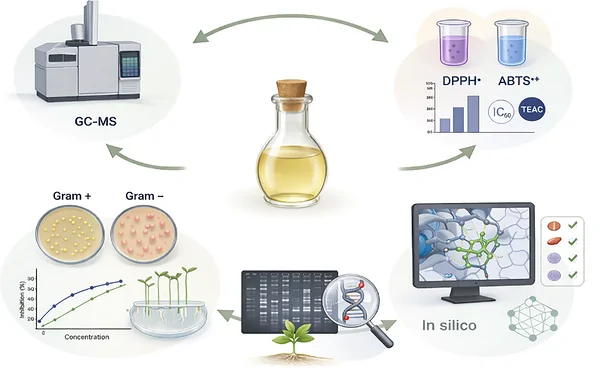

## 1 Introduction

Humans have used medicinal and aromatic plants since prehistoric times. Initially used in mystical rituals, medicinal, and aromatic plants were discovered for their flavoring and healing properties. Nowadays, they are fundamental to human life, serving as spices, cosmetics, textiles, and especially sources of medicines [1]. In many regions, including America, Africa, and Asia, over 85 % of people primarily use herbal medicine for healthcare. Growing concerns about the adverse side effects of synthetic drugs have increased global demand for natural, plant‐based remedies. Consequently, the global herbal supplement market reached around US$115 billion by 2020 [2].

Essential oils are one of the most valuable products obtained from medicinal and aromatic plants through distillation techniques [3]. The essential oils are complex mixtures of mostly terpenes, phenols, alcohols, ketones, esters, and aromatic compounds, with a few dominant molecules accounting for most of the content [4]. Their rich chemical diversity and the quantity of their compounds give them various biological effects, including antioxidant, antibacterial, antiviral, antifungal, antimutagenic, antigenotoxic, and anti‐inflammatory properties [5, 6]. However, the composition of essential oils varies depending on environmental conditions, such as geographical region, soil type, climate, altitude, plant stress, and extraction methods [7]. Hence, it is a scientifically justified approach to re‐evaluate the composition and biological activities of the essential oil of a plant grown in a different geographical region, even if the species has been researched before.

The genus *Origanum* is an important aromatic and medicinal plant genus belongs to the family Lamiaceae, tribe Mentheae. It comprises approximately 43 species, including six subspecies and 18 natural hybrids, which are mainly distributed in the Mediterranean, Euro‐Siberian, and Iranian‐ Turanian phytogeographical regions [8]. Approximately 75% of the Origanum taxa occur in the Western Mediterranean region, and 16 species are recognized as endemic to Türkiye [9] highlighting the country as an important center of diversity for this genus. *Origanum* species are known as ‘Oregano’ (Kekik) and is used in traditional medicine as well as in culinary and industrial applications [10]. According to previous studies, *Origanum* species exhibit a broad spectrum of biological activities, including antibacterial [11], antifungal [12], antioxidant [13], antispasmodic [14], antitumoral [15], expectorant [16], antiparasitic, anti‐helminthic [17], carminative [18], diaphoretic, stimulant [19], anti‐inflammatory [20], antidiabetic [21], and analgesic [22] properties. In traditional medicine, these plants have been used to treat cough, colic, toothache, rheumatism, and menstrual disorders [23]. In addition, *Origanum* species are widely utilized as natural disinfectants, fragrance additives in perfumes and soaps, and flavoring agents in foods and beverages [24] reflecting their economic and pharmacological importance.

Among these species, *Origanum minutiflorum* [25], commonly known as “toga kekik” (toga thyme) or “dağ kekiği” (mountain thyme), is an endemic taxon primarily distributed in the eastern Mediterranean and southwestern Türkiye [10]. There has been much research about the chemical composition and biological activities of the essential oils derived from *O. minutiflorum* due to their rich content of bioactive volatile compounds [3, 10, 26, 27]. However, despite its endemic status and potential pharmacological importance, *O. minutiflorum*, remains comparatively under‐investigated, and only a limited number of studies have reported its chemical composition and biological properties [28]. Essential oils extracted from *Origanum* species have attracted considerable attention due to their potential applications in pharmaceuticals, food preservation, and cosmetics [29, 30]. Among the analytical techniques used to investigate essential oils, Gas chromatography coupled with mass spectrometry (GC‐MS) is a powerful analytical tool used extensively to characterize the chemical composition of essential oils, enabling detailed identification and quantification of volatile constituents [31, 32]^.^

In recent years, computational approaches have increasingly complemented phytochemical analyses. Several in silico studies involving different *Origanum* species (e.g., *O. majorana*, *O. vulgare*, *O. elongatum*) have combined GC–MS profiling with molecular docking, ADMET prediction, and occasionally molecular dynamics (MD) simulations to propose possible molecular mechanisms underlying antimicrobial or anticancer activities [33]. Nevertheless, comparable computational investigations focusing specifically on *O. minutiflorum* remain scarce, representing an important knowledge gap in literature [34, 35, 36]. Available studies on *O. minutiflorum* mainly report chemical composition analyses and in vitro biological activity evaluations, while only a limited number of investigations have examined the molecular interactions of its major constituents with biological targets through docking approaches [37]. Therefore, a targeted literature assessment was conducted for the major constituents of *O. minutiflorum* evaluated in the present study. Among these constituents, *p*‐cymene has previously been investigated in molecular docking studies, particularly in half‐sandwich ruthenium–*p*‐cymene‐derived metalloantibiotics, where docking against MurB (PDB: 1HSK) and DNA gyrase A revealed binding energies of approximately −12 kcal/mol and −6.3 kcal/mol, respectively [38, 39, 40]. In contrast, both *γ*‐clausenane and borneol, despite their prominence as major constituents in many essential oils (including our study's *O. minutiflorum sample*), appear absent in publicly indexed docking literature for the bacterial enzyme targets we consider (MurB 1HSK; FabZ 1U1Z; glycerophosphodiesterase 2DXN; thymidine kinase 2JA1; L‐methionine γ‐lyase 3JWB; transcriptional regulator TcaR 3KP3; DNA gyrase A 4DDQ; and DNA topoisomerase IV 6WAA), signifying a clear gap and opportunity for novel in silico exploration.

Accordingly, the novelty of the present study lies not in the first characterization of *O. minutiflorum* essential oil, but in its integrated evaluation combining region‐specific chemical profiling, antioxidant and antibacterial activities, RAPD‐PCR‐based genotoxicity assessment, and in silico ADME and molecular docking analyses of its major volatile constituents. This combined experimental and computational approach provides a broader evaluation of the biological potential and safety‐related properties of *O. minutiflorum* essential oil.

This study aimed to determine the essential oil yield and chemical composition of *O. minutiflorum* collected from Diyarbakır province using GC–MS analysis. In addition, in silico investigations—including ADME prediction, molecular docking, and short molecular dynamics simulations—were performed for the major constituents of the essential oil. Furthermore, the total phenolic content (TPC) as well as the antioxidant, antibacterial, and genotoxic activities of the essential oil were evaluated.

## 2 Results and Discussion

### 2.1 GC‐MS Analyses of OmEO

The OmEO was subjected to GC‐MS analysis using an Rxi‐5MS capillary column. Component identification was performed by comparing mass spectra against standard libraries (FFNSC1.2 and W9N11) and further validated by consulting literature sources [31, 41, 42]. Table 1 summarizes the chemical composition, relative percentages, and GC retention indices of the analyzed essential oil.

**TABLE 1:** Identified essential oils (EOs) of *O. minutiflorum*.

No	Compounds	RI^a^	Lit RI	RT^b^	Area (%)^c^
1	Hex‐3(E)‐enol	849	847	10.101	0.1
2	2,5‐Diethyl‐tetrahydrofuran	902	900	11.768	0.1
3	*α*‐Pinene	933	933	13.204	2.8
4	*δ*‐3‐Carene	942	948	13.624	3.8
5	Camphene	950	946	14.254	0.5
6	*ß*‐Pinene	978	974	15.679	3.1
7	*ß*‐Myrcene	993	992	16.107	1.4
8	*p*‐Cymene	1029	1022	18.483	36.7
9	Borneol	1125	1125	20.840	10.6
10	Terpinen‐4‐ol	1206	1192	26.428	3.9
11	Thymol	1303	1305	32.326	4.0
12	*γ*‐Clausenane	1477	1470	35.171	31.1
13	Spathulenol	1600	1580	45.557	1.0
				Total	99.1

^a^ Retention index calculated from retention times relative to n‐alkane series (C6 –C30) on the non‐polar Rxi‐5MS column.

^b^ RT: Retention Time.

^c^ Percentages obtained by FID peak‐area normalization.

**FIGURE 1:**
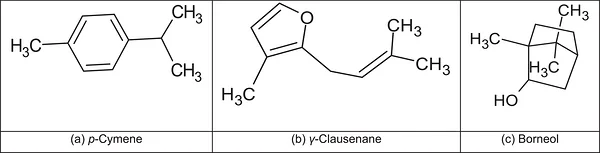
Chemical structures of the major constituents identified in OmEO.

In the OmEO, thirteen components were identified, making up 99.1% of the total composition. The predominant compounds were *p*‐Cymene, *γ*‐Clausenane, and Borneol, present at concentrations of 36.7%, 31.1%, and 10.6%, respectively. Table 1 details the observed diversity in these essential oil constituents. The chemical structures of the major constituents are presented in Figure 1. The essential oil of the studied plant consisted of 13 identified constituents, accounting for 99.1% of the total oil composition. These compounds were categorized into several chemical classes, including monoterpene hydrocarbons (11.6%), oxygenated monoterpenes (18.5%), sesquiterpene hydrocarbons (31.1%), oxygenated sesquiterpenes (1.0%), aromatic monoterpenes (36.7%), alcohols (0.1%), and oxygenated aliphatic compounds (0.1%) (Table 2). Among the monoterpene hydrocarbons, *α*‐Pinene (2.8%), *δ*‐3‐Carene (3.8%), *ß*‐Pinene (3.1%), and *ß*‐Myrcene (1.4%) were notable contributors. The aromatic monoterpene fraction was predominantly represented by *p*‐Cymene (36.7%), while oxygenated monoterpenes included Borneol (10.6%), Terpinen‐4‐ol (3.9%), and Thymol (4.0%). *γ*‐Clausenane constituted the major sesquiterpene hydrocarbon at 31.1%, and Spathulenol represented the oxygenated sesquiterpenes at 1.0%. Minor constituents such as Hex‐3(E)‐enol (0.1%) and 2,5‐Diethyl‐tetrahydrofuran (0.1%) were detected in the alcohol and oxygenated aliphatic compound classes, respectively. The detailed retention indices (RI), retention times (RT), and relative peak areas are presented in Table 1. These findings highlight the chemical diversity and richness of the essential oil, with aromatic monoterpenes and sesquiterpene hydrocarbons being the dominant classes of compounds. Recent studies have highlighted the importance of comprehensive chemical profiling in understanding the composition and biological properties of plant‐derived products [43, 44]. The chemical composition of essential oils may also contribute to their biological activities [45].

**TABLE 2:** Composition of chemical classes in OmEO.

Chemical classes	%^a^	NC^b^
Monoterpene hydrocarbons	11.6	5
Oxygenated monoterpenes	18.5	3
Sesquiterpene hydrocarbons	31.1	1
Oxygenated sesquiterpenes	1	1
Aromatic monoterpene	36.7	1
Alcohols	0.1	1
Oxygenated aliphatic compounds	0.1	1
Total	99.1	13

^a^ Percentages obtained by FID peak‐area normalization.

^b^ Number of compounds.

When compared with the literature, the OmEO composition in this study shares common major constituents such as *p*‐Cymene and Borneol, corroborating previous research [28, 46]. However, the relatively high content of *γ*‐Clausenane observed here contrasts with some reports, which may be due to environmental factors or methodological differences. This highlights the chemical variability within the species and underscores the importance of region‐specific analyses. It is well established that essential oil yield and composition are affected by seasonal variations, climatic conditions, geographical factors, distillation techniques, and harvesting time, as supported by previous studies [27, 47].

### 2.2 Antioxidant Activity and Total Phenolic Content (TPC) of OmEO

In this study, three complementary antioxidant assays based on different reaction mechanisms DPPH•, ABTS•^+^, and FRAP were employed to evaluate the antioxidant potential of OmEO. In addition, TPC was determined to estimate the abundance of bioactive phenolic constituents in the obtained oil. Table 3 presents the antioxidant activity and TPC results of OmEO in comparison with the standard compounds. The radical scavenging activities of OmEO, evaluated using DPPH• and ABTS•^+^ assays, yielded IC_50_ values of 1.0864 ± 0.0296 mg/mL and 0.0055 ± 0.0002 mg/mL, respectively. OmEO exhibited significantly lower DPPH• radical scavenging activity (IC_50_ = 1.0864 ± 0.0296 mg/mL) compared to the reference antioxidant BHT (IC_50_ = 0.0143 ± 0.0004 mg/mL). The ABTS•^+^ radical scavenging activity of OmEO (IC_50_ = 0.0055 ± 0.0002 mg/mL) was slightly lower than that of the reference antioxidant Trolox (IC_50_ = 0.0032 ± 0.0000 mg/mL), indicating moderate antioxidant potential. OmEO exhibited a FRAP value of 745.56 ± 1.47 µM TEAC, indicating a considerable reducing capacity. Recent studies have also highlighted the antioxidant potential of plant‐derived essential oils and their bioactive constituents [43, 45]. This effect may be attributed to the ability of OmEO antioxidants to reduce Fe^3^^+^ ions through electron‐donating mechanisms. The TPC of OmEO was quantified as 558.57 ± 1.43 µg/mL gallic acid equivalents (GAE). OmEO contains substances containing the Folin–Ciocalteu reagent, which is confirmed by the presence of the polar phenolic component.

**TABLE 3:** TPC and antioxidant activity of OmEO^a^ and standards.

		Antioxidant activity^a^		
Sample	TPC^b^ (GAE, µg/mL)	DPPH•scavenging (IC_50_, mg/mL)	ABTS•^+^ radical scavenging (IC_50_, mg/mL)	FRAP^b^ (TEAC, µM)
OmEO	558.57 ± 1.43	1.0864 ± 0.0296	0.0055 ± 0.0002	745.56 ± 1.47
Trolox	NT	0.0028 ± 0.0000	0.0032 ± 0.0000	#
BHT	NT	0.0143 ± 0.0004	0.0005 ± 0.0000	NT
Quercetin	#	NT	0.0015 ± 0.0000	NT
Gallic Acid	#	NT	0.0007 ± 0.0001	NT

Abbreviations: ABTS•^+^,2,2’‐azino‐bis (3‐ethylbenzothiazoline‐6‐sulfonic acid); BHT, butylated hydroxytoluene;DPPH•, 2,2‐diphenyl‐1‐picrylhydrazyl; FRAP, Ferric reducing antioxidant power; GAE, gallic acid equivalent; IC_50_, Antioxidant concentration causing 50% radical scavenging; TEAC, trolox equivalent to antioxidant capacity;TPC, Total phenolic content.

^a^ Test results were expressed as mean ± standard error (SD) of three experiments (*p* < 0.01).

^b^ OmEO was tested at a concentration of 5 mg/mL.

NT: Not tested.

#:Trolox was used to constructing a calibration curve used for the calculation of TEAC values and gallic acid and quercetin were used to obtain TPC values.

Although the antioxidant activity of various solvent extracts of *O. minutiflorum* has been extensively investigated, limited studies have addressed the antioxidant activity and total phenolic content of its essential oil. The literature review revealed that the antioxidant activity, TPC, and chemical composition of the aqueous extracts of seven members of the Lamiaceae family, including Toka oregano (*Origanum minutiflorum* [25], have previously been reported [48]. According to the results, the aqueous extract of *O. minutiflorum* exhibited moderate antioxidant activity and phenolic content compared to the other extracts evaluated. In their study, Özcan and Özcan [49] evaluated and compared the DPPH radical scavenging activities of extracts prepared by Soxhlet and ultrasonic water bath extraction methods employing various ratios of methanol, acetone, water, and acetic acid as extraction solvents. According to the findings of [50], the methanol extract of *O. minutiflorum* exhibited moderate free radical scavenging activity. The results obtained from the essential oil in the present study are consistent with those previously reported for solvent extracts. Sokmen, et al. [51] reported that the essential oil of *O. minutiflorum* exhibits weak free radical scavenging activity. Overall, the findings suggest that OmEO demonstrates considerable potential with respect to its in vitro antioxidant activity. However, further in vivo studies and detailed mechanistic investigations are required to confirm its efficacy and safety.

### 2.3 Evaluation of Antimicrobial Activity

In this study, the OmEO was tested against four Gram‐negative (*K. pneumoniae*, *C. freundii*, *E. aerogenes*, *P. aeruginosa*) and four Gram‐positive (*S. aureus*, *B. cereus*, *B. subtilis*, *S. epidermidis*) at doses ranging from 400 to 25 µg/mL. According to the results, the studied oil exhibited significant antibacterial activity against both Gram‐negative and Gram‐positive bacteria, varying with the dose. The bacteria were affected more seriously by *O. munitiflorum*’s oil at high‐dose oil applications. The MIC was determined to be 50 µg/mL against all tested bacterial strains, with the exception of *Enterobacter aerogenes*, for which the MIC was 100 µg/mL (Table 4). There are many reports about the antimicrobial activity of *O. munitiflorum*’s oil against various bacteria (*S. aureus*, *S. faecalis*, *B. subtilis*, *E. coli*, *L. monocytogenes*, *S. typhimurium*, ciprofloxacin‐resistant *Campylobacter* spp., *A. hydrophila*, *Y. enterocolitica*, *K. pneumonia*, *P. mirabilis*, *B. cereus*, MRSA, *S. pneumoniae*, *S. enteritidis*, *C. albicans*, *A. parasiticus*, *A. flavus*, *E. cloacae*, *C. albicans*, *C. tropicalis*, and *T. glabrata* [3, 27, 28, 46, 52, 53, 54], and phytopathogenic bacteria [55]. The antibacterial results of this study were supported by the literature. Essential oils, complex mixtures of terpenoids and various other constituents, exhibit antimicrobial activity thanks to these constituents, especially terpenes and terpenoids. They display antimicrobial activity through disrupting cell membranes, inhibiting gene expression, and enzyme activity [56]. According to the GC‐MS analyses, the main components of the essential oil were *p*‐Cymene, *γ*‐Clausenane, and Borneol, which have been reported as antibacterial compounds. *p*‐Cymene, a monoterpene hydrocarbon, was reported to have moderate antibacterial activity against both Gram‐positive and Gram‐negative bacteria through disruption of cell membrane integrity. Besides, *p*‐Cymene exhibits strong synergistic effects when combined with phenolic compounds such as carvacrol, significantly enhancing bactericidal efficacy against different bacteria [48, 57, 58, 59]. Borneol, a bicyclic monoterpene alcohol, has been found to exhibit antibacterial and antibiofilm effects, primarily through its interference with membrane fluidity and bacterial permeability. Borneol also enhances the membrane permeability and facilitates the uptake and potency of other antimicrobial agents [60, 61]. Although *γ*‐clausenane was the second most abundant constituent of OmEO, its specific contribution to the antibacterial activity of the essential oil remains unclear and requires further investigation. Recent research has also highlighted the biological potential of plant‐derived essential oils and their complex mixtures of bioactive constituents [45].

**TABLE 4:** Antibacterial activity of OmEO.

Bacteria	400 µg/mL	300 µg/mL	200 µg/mL	100 µg/mL	50 µg/mL	25 µg/mL	PC	NC	MIC
*Klebsiella pneumoniae*	20.33 ± 0.33^g^	15.66 ± 0.66^e^	12.66 ± 0.33^d^	9.66 ± 0.33^c^	8.33 ± 0.33^b^	0 ±.0^a^	18.0 ± 0.57^f^	0 ± 0^a^	50
*Citrobacter freundii*	20.33 ± 0.66^f^	16.33 ± 0.33^e^	11.66 ± 0.33^d^	9.0 ± 0^c^	7.0 ±.057^b^	0 ± 0^a^	17.0 ± 0.57^e^	0 ± 0^a^	50
*Enterobacter aerogenes*	18.66 ± 0.33^f^	12.00 ± 0^d^	10.0 ± 0.57^c^	7.0 ± 0.57^b^	0 ± 0^a^	0 ± 0^a^	16.33 ± 0.33^e^	0 ± 0^a^	100
*Pseudomonas aeruginosa*	18.0 ± 0.57^e^	14.66 ± 0.33^d^	10.33 ± 0.33^c^	7.0 ± 0^b^	0 ± 0^a^	0 ± 0^a^	14.66 ± 0.66^d^	0 ± 0^a^	50
*Staphylococcus aureus*	18.66 ± 0.33^f^	16.33 ± 0.66^e^	11.66 ± 0.66^d^	10.0 ± 0.57^c^	8.0 ± 0.57^b^	0 ± 0^a^	18.66 ± 0.33^f^	0 ± 0^a^	50
*Bacillus cereus*	22.66 ± 0.66^g^	16.33 ± 0.33^e^	14.33 ± 0.33^d^	10.0 ± 0^c^	7.33 ± 0.57^b^	0 ± 0^a^	20.33 ± 0.57^f^	0 ± 0^a^	50
*Bacillus subtilis*	24.33 ± 0.33^f^	20.0 ± 0.57^e^	14.33 ± 0.66^d^	10.0 ± 0.57^c^	7.66 ± 0.33^b^	0 ± 0^a^	21.0 ± 0^e^	0 ± 0^a^	50
*Staphylococcus epidermidis*	25.33 ± 0.33^f^	20.33 ± 0.33^e^	14.0 ± 0^d^	10.33 ± 0.33^c^	6.66 ± 0.33^b^	0 ± 0^a^	19.66 ± 0.33^e^	0 ± 0^a^	50

In the same row, values indicated with the same letter do not have a significant difference at the *p* < 0.05 level according to Duncan's multiple range test.

abbreviations: CHL, chloramphenicol; Con, concentration; ERY, erythromycin; NC, negative control (DMSO); PC, positive control; TET, tetracycline.

In conclusion, OmEO is a great source of antimicrobial compounds. The compounds are able to show antimicrobial activity individually, but when combined, they can show a stronger antimicrobial effect through synergistic interaction. These results are thought to contribute to researches on drug components in the future.

### 2.4 RAPD‐PCR Results and Morphological Parameters

**FIGURE 2:**
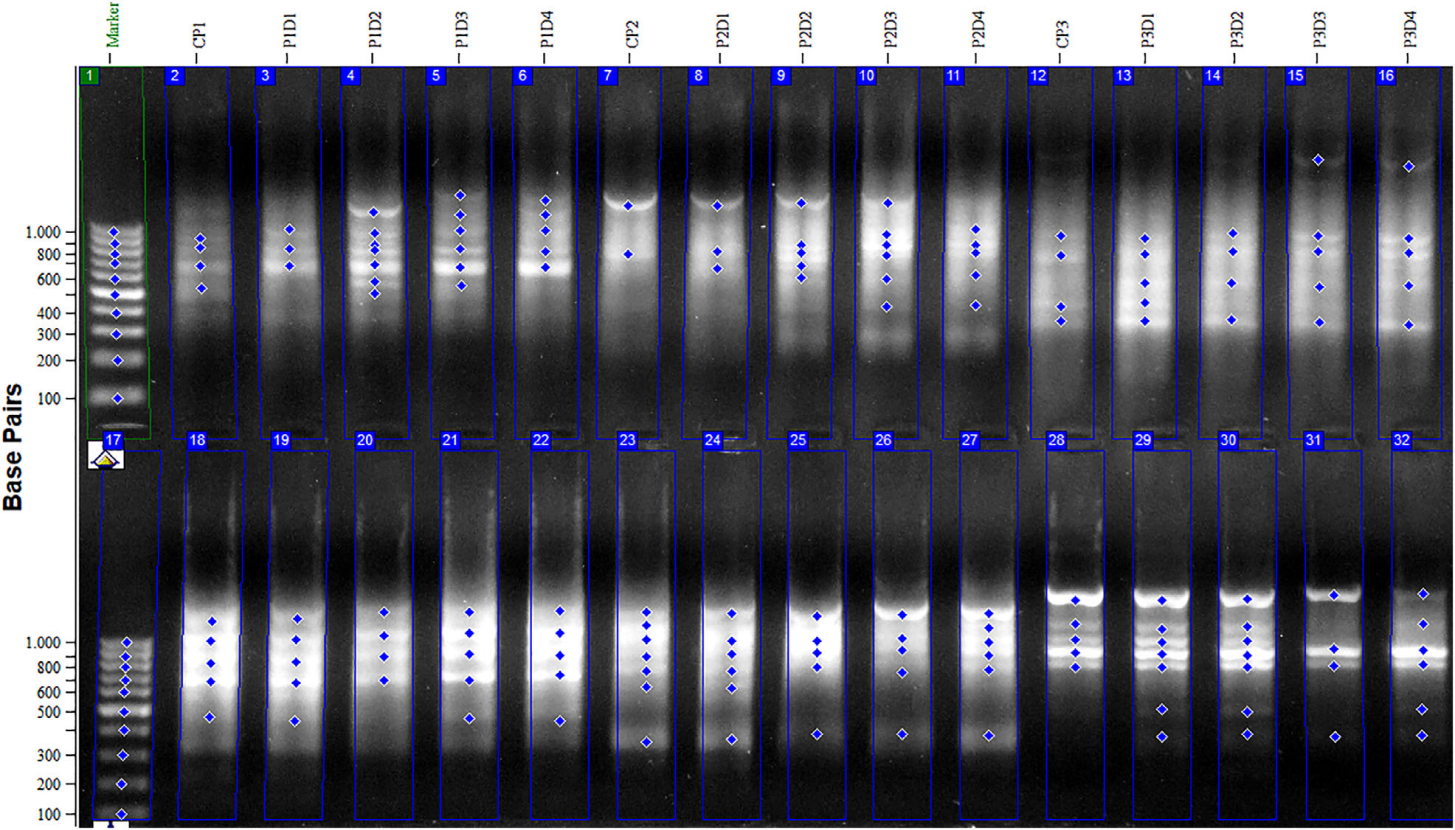
RAPD profiles of genomic DNA extracted from *Triticum aestivum* seedlings exposed to OmEO with primer 1–6 (C: Control, P: Primer, D: Dose).

**FIGURE 3:**
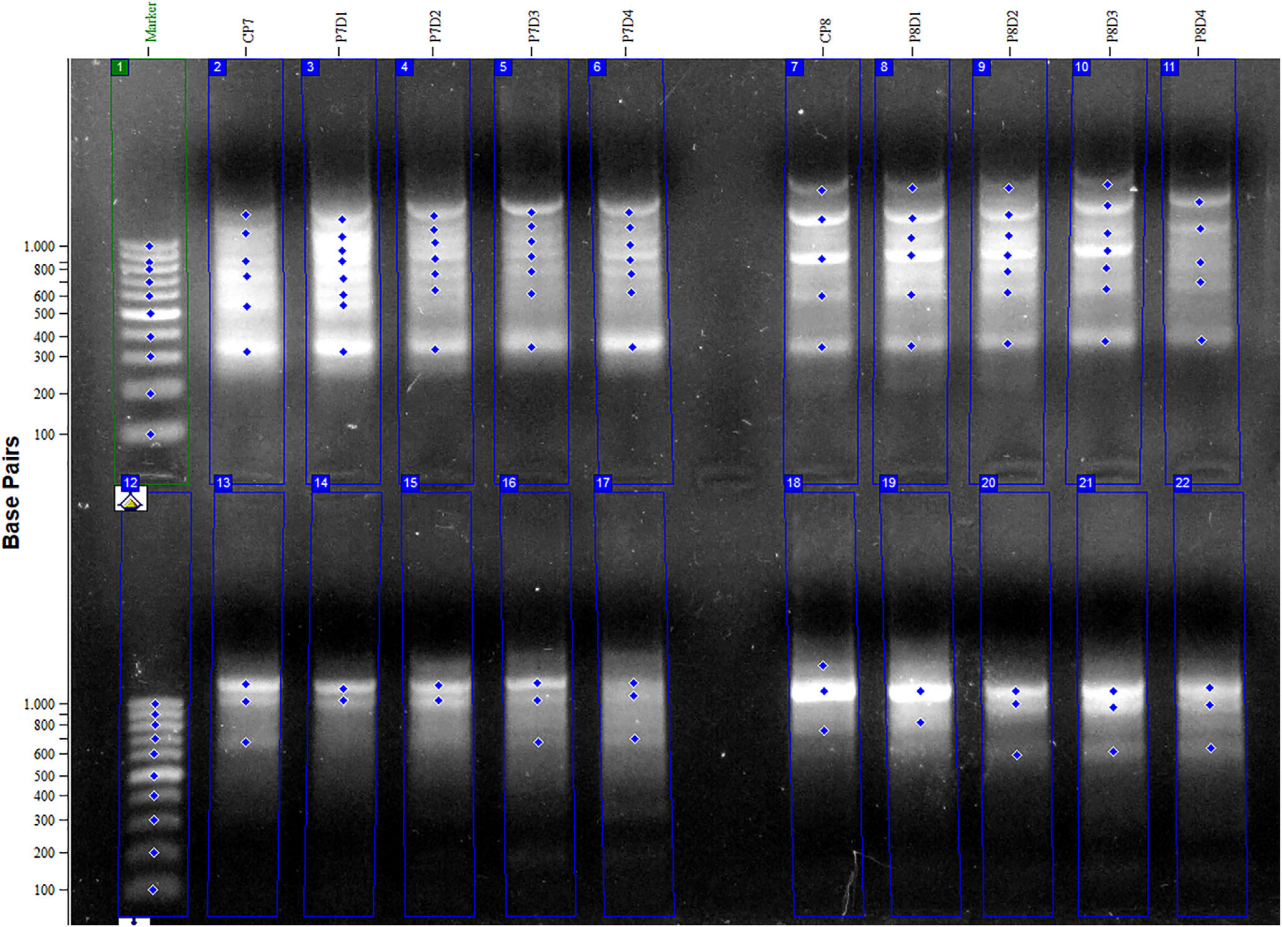
RAPD profiles of genomic DNA extracted from *Triticum aestivum* seedlings exposed to OmEO with primer 7–10 (C: Control, P: Primer, D: Dose).

The molecular analysis revealed dose‐dependent alterations in DNA banding patterns of *Triticum aestivum* treated with OmEO (Table 5). The polymorphism ratio increased progressively with the concentration of the treatment, rising from 26.66% at 0.2 µL/mL to 60% at both 0.8 and 1.6 µL/mL. This increase indicates enhanced genomic alterations induced by higher doses. The alterations in the PCR banding patterns of wheat samples amplified with ten different primers are presented in Figures 2 and 3. As illustrated in Figures 2 and 3, OmEO treatment resulted in a reduction in DNA band intensity compared with the control group. Furthermore, the disappearance of existing bands and the appearance of novel bands were detected, suggesting OmEO‐induced changes in genomic DNA profiles.

**TABLE 5:** The number and molecular size of polymorphic bands for all primers in the OmEO treated *Triticum aestivum* seedlings.

Primer	*Triticum aestivum*					
	Control		0.2 µL/mL	0.4 µL/mL	0.8 µL/mL	1.6 µL/mL
**1**	4	+	ND	1166,1022	1266,1166,1022,508	1266,1166,1022
		—	944,542		944	944,542
**2**	2	+	658	888,658,607	1022,888,607,433	1022,888,607,433
		—	ND	ND	ND	1222
**3**	4	+	576	576	1611,576	1611,576
		—	ND	433	433	433
**4**	5	+	ND	ND	ND	ND
		—	ND	ND	472	ND
**5**	7	+	ND	ND	ND	ND
		—	1118	1118,643	1118,643	643
**6**	5	+	512,368	512,368	368	512,368
		—	ND	ND	1127,1018	ND
**7**	6	+	984,608	984,608	984,608	984,608
		—	ND	537	537	537
**8**	5	+	1055	1055,900	1055,900	1055,900
		—	ND	ND	ND	1374,935
**9**	3	+	ND	ND	ND	ND
		—	679	679	ND	ND
**10**	3	+	ND	1000,637	1000,637	1000,637
		—	1366	1366,760	1366,760	1366,760
**Total band**	45		12	21	27	27
**Polymorphism value**			26.66	46.66	60	60
**GTS Ratio**			73.33	53.33	40	40

Conversely, the Genomic Template Stability (GTS) values showed a consistent decrease, from 73.33% at 0.2 µL/mL to 40% at the highest concentrations. The marked reduction in GTS suggests that elevated doses of the essential oil may induce significant genotoxic effects in *T. aestivum* seedlings by compromising genome stability.

Plants, which lack both active mobility and an adaptive immune system comparable to that of animals, rely predominantly on chemical defense strategies, particularly the production of allelochemicals, to ensure their survival. Among these, essential oils represent some of the most potent agents, exhibiting defensive activity against microorganisms, competing plants, and even herbivorous animals. Their natural origin has facilitated widespread application across diverse fields. However, potential toxic effects associated with these compounds may impose limitations on their use [62]. In the present study, increasing doses were observed to induce genotoxic effects, as evidenced by alterations in Genomic Template Stability (GTS) ratios. This observation is consistent with findings reported in previous studies. For instance, it has been demonstrated that these secondary metabolites can exert cytotoxic effects on human lymphocyte cell lines, leading to increased chromosomal aberrations and elevated micronucleus formation [63]. Similarly, another study reported that essential oils may exhibit low‐hazard acute oral toxicity while still possessing mutagenic potential on germ cells [64].

The effects of different doses of OmEO on the root and stem lengths of *Triticum aestivum* seedlings are presented in Table 6. While root lengths remained statistically similar across all treatments (*p* > 0.05), stem lengths were significantly affected by increasing concentrations of the essential oil (*p* < 0.05). The control group exhibited the longest stem length, which was significantly higher than all other treatments. At 0.2 and 0.4 µL/mL doses, the stem lengths decreased respectively, with no significant difference. The most substantial reduction was observed at the highest concentrations, with stem lengths forming a separate statistical group (*p* < 0.05). These findings indicate that OmEO exerts a dose‐dependent inhibitory effect on stem elongation, whereas root growth appears to be less sensitive to treatments.

**TABLE 6:** Root and shoot lengths of *Triticum aestivum* seeds treated with OmEO.

Doses	Root length	Stem length
Control	0.6 ± 0.02^a^	7.06 ± 0.64 ^a^
0.2 µL/mL	0.56 ± 0.01^a^	3.28 ± 0.57^b^
0.4 µL/mL	0.57 ± 0.01^a^	3.37 ± 0.49^b^
0.8 µL/mL	0.55 ± 0.01^a^	1.11 ± 0.22^c^
1.6 µL/mL	0.55 ± 0.02^a^	0.52 ± 0.16^c^

Values followed by the same letter in the same row are not significantly different (*p* < 0.05; Duncan's test).

Furthermore, the application of essential oils, either through direct spraying onto germinating seeds or onto seedlings [65], has been shown to reduce germination rates and, after 24 h, to increase the presence of necrotic plant tissues [66]. Owing to these phytotoxic properties, essential oils have been recognized as natural compounds with potential utility in the control of invasive or undesirable plant species [65, 66, 67]. A similar effect was observed in the present study, where the impacted regions of the plant were predominantly the above ground tissues. This suggests that the applied essential oils are absorbed through the roots and subsequently exert their effects after internal translocation within the plant. It is proposed that allelochemicals may disrupt osmoregulatory processes, thereby inducing stress conditions in the plant and ultimately constraining its growth and development [68].

### 2.5 Computational Studies

#### 2.5.1 In Silico ADME Analysis

The pharmacokinetic and drug‐likeness profiles of the selected volatile constituents Borneol, *γ*‐Clausenane, and *p*‐Cymene were evaluated using in silico ADME prediction tools, and the results are summarized in Table 7. The assessment provides key parameters such as Lipinski's rule compliance, topological polar surface area (TPSA), partition coefficient (Log P), gastrointestinal (GI) absorption, blood–brain barrier (BBB) permeability, and cytochrome P450 (CYP) interaction patterns. All three compounds fulfilled Lipinski's rule of five, indicating favorable oral bioavailability and molecular properties consistent with drug‐like behavior. However, *p*‐Cymene exhibited a single violation due to its relatively high lipophilicity (MLOGP > 4.15), a feature frequently observed among small, hydrophobic terpenes. Despite this, its physicochemical properties remain within acceptable ranges for potential lead optimization. The TPSA values for the molecules ranged from 0.00 Å^2^ (*p*‐Cymene) to 20.23 Å^2^ (Borneol), suggesting low polarity and, consequently, good passive membrane permeability. These results correlate with the predicted GI absorption, which was classified as high for Borneol and *γ*‐Clausenane, but low for *p*‐Cymene, likely due to its extreme hydrophobicity and absence of hydrogen bond donors or acceptors. All compounds were predicted to be BBB permeant, indicating their potential to cross the central nervous system (CNS) barrier. This property may contribute to both their pharmacological activity and possible neuroactive effects, consistent with prior reports on monoterpenes and their CNS modulatory behavior.

**TABLE 7:** SwissADME properties of Borneol, *γ*‐Clausenane, and *p*‐Cymene.

Compound	Borneol	*γ*‐Clausenane	*p*‐Cymene
Lipinski	Yes	Yes	Yes (1 violation: MLOGP>4.15)
TPSA (Å^2^)	20.23	13.14	0.00
Consensus Log (Po/w)	2.38	2.85	3.50
Bioavailability Score	0.55	0.55	0.55
GI Absorption	High	High	Low
BBB permeant	Yes	Yes	Yes
P‐gp substrate	No	No	No
CYP2D6	No	No	Yes

None of the tested compounds were identified as P‐glycoprotein (P‐gp) substrates, suggesting that efflux mechanisms are unlikely to limit their intracellular accumulation. Moreover, CYP2D6 inhibition was predicted only for *p*‐Cymene, implying a moderate risk of metabolic interference, while Borneol and *γ*‐Clausenane appear to possess safer metabolic profiles.

The bioavailability scores were identical (0.55) for all three compounds, indicating moderate systemic exposure potential after oral administration. When considered alongside their Log *p* values (2.38–3.50), these findings suggest an optimal balance between solubility and permeability for Borneol and *γ*‐Clausenane, whereas *p*‐Cymene's higher lipophilicity could limit aqueous solubility but enhance membrane diffusion.

Overall, the in silico ADME analysis supports the drug‐likeness and pharmacokinetic feasibility of the selected terpenoid compounds. Particularly, Borneol and *γ*‐Clausenane exhibited balanced lipophilicity, favorable absorption characteristics, and minimal metabolic liabilities‐making them promising scaffolds for further biological and molecular studies targeting DNA gyrase A and related bacterial enzymes.

In silico analyses performed on the major terpenoid constituents of OmEO namely Borneol, *γ*‐Clausenane, and *p*‐Cymene revealed promising pharmacokinetic and molecular interaction profiles supporting their bioactive potential. ADME predictions indicated that all three compounds complied with Lipinski's rule of five, suggesting favorable oral bioavailability, acceptable lipophilicity, and low predicted toxicity. These properties are consistent with previous studies demonstrating that small monoterpenes typically exhibit favorable drug‐likeness profiles, high membrane permeability, and efficient passive diffusion across lipid bilayers due to their moderate lipophilicity and low molecular weight [69, 70]. Recent studies have emphasized the value of pharmacokinetic and bioavailability assessments in evaluating the therapeutic potential of bioactive natural compounds [71]. In addition, integrated in silico approaches, including target prediction and molecular docking, have been increasingly used to explore the potential mechanisms of natural compounds [72, 73].

### 2.6 Molecular Docking

Molecular docking simulations were performed to predict the binding affinities and interaction modes of Borneol, γ‐Clausenane, and *p*‐Cymene against a panel of bacterial target proteins, as listed in Table 8. The docking scores (binding free energies, kcal/mol) were compared, where applicable, with the scores obtained by re‐docking the corresponding co‐crystallized ligands under the same AutoDock Vina conditions to contextualize the relative binding profiles of the naturally derived compounds.

**TABLE 8:** Molecular docking scores (kcal/mol) of the study ligands and redocked co‐crystallized ligands used for protocol validation.

PDB Code	Borneol	*γ*‐Clausenane	*p*‐Cymene	Redocked co‐crystallized ligand
1HSK	−5.9	−6.3	−6.7	—
1U1Z	−3.8	−4.7	−4.1	—
2DXN	−5.0	−4.6	−4.8	—
2JA1	−5.7	−5.6	−5.8	TTP (−6.3)
3JWB	−5.3	−5.2	−5.3	NLE (−3.8)
3KP3	−4.5	−4.4	−4.7	AIC (−5.2)
4DDQ	−4.8	−4.8	−5.0	—
6WAA	−5.1	−5.0	−5.1	TNJ (−6.8)

Overall, the ligands demonstrated moderate to strong affinity toward the tested proteins, with docking energies ranging from −3.8 to −6.7 kcal/mol. Among them, *p*‐Cymene generally exhibited the lowest (most negative) binding energies across multiple receptors, suggesting a slightly higher binding propensity compared to Borneol and *γ*‐Clausenane. For instance, *p*‐Cymene yielded docking scores of −6.7, −5.8, and −5.0 kcal/mol for 1HSK, 2JA1, and 4DDQ, respectively—values comparable to or even surpassing those of several reference ligands in similar bacterial docking studies.

**FIGURE 4:**
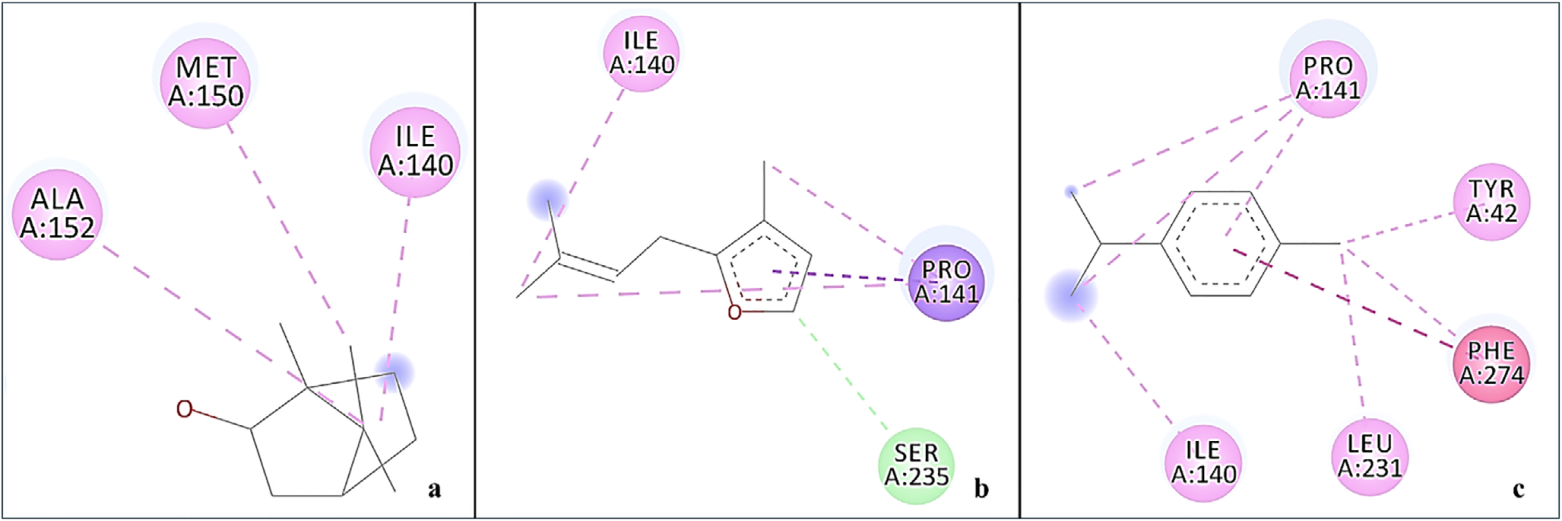
2D interaction diagram of ligands‐MurB (PDB ID: 1HSK) enzyme complex illustrating key non‐covalent interactions. Light pink dashed lines represent π–alkyl interactions, purple lines indicate π‐sigma interactions, and dark pink lines denote π–π T‐shaped interactions. Light green lines correspond to carbon–hydrogen bonds, while dark green lines represent conventional hydrogen bonds. (a) Borneol, (b) *γ*‐Clausenane, (c) *p*‐Cymene.

The 1HSK (MurB) enzyme, which plays a critical role in bacterial cell wall biosynthesis, exhibited the most favorable interactions among all receptors, with *γ*‐Clausenane and *p*‐Cymene displaying binding affinities of −6.3 and −6.7 kcal/mol, respectively. RCSB PDB identifies FAD as the crystallographic small molecule in 1HSK; because FAD is a bound redox cofactor rather than a pharmacological inhibitor, its docking score should be interpreted as a structural pocket reference rather than as a direct inhibitory comparator. All three study compounds were accommodated within the FAD‐associated region (Figure 4). Borneol exhibited contacts involving ALA152, ILE140, and MET150, whereas *γ*‐Clausenane was stabilized predominantly by nonpolar/π‐type contacts around ILE140 and PRO141. *p*‐Cymene established multiple π–alkyl and π–π T‐shaped interactions with residues including TYR42, PRO141, PHE274, and LEU231. Accordingly, the binding of the two hydrocarbon ligands, *γ*‐Clausenane and *p*‐Cymene, is interpreted primarily in terms of hydrophobic and π‐mediated stabilization rather than conventional hydrogen bonding.

The 2JA1 (Thymidine kinase) and 6WAA (DNA topoisomerase IV) receptors also exhibited notable interactions with all three compounds, with average binding energies of approximately −5.5 kcal/mol. These targets are key in bacterial DNA replication and nucleotide metabolism, and the observed affinities support the hypothesis that the studied terpenoids may interfere with nucleic acid processing enzymes through non‐covalent stabilization of the binding pocket.

**FIGURE 5:**
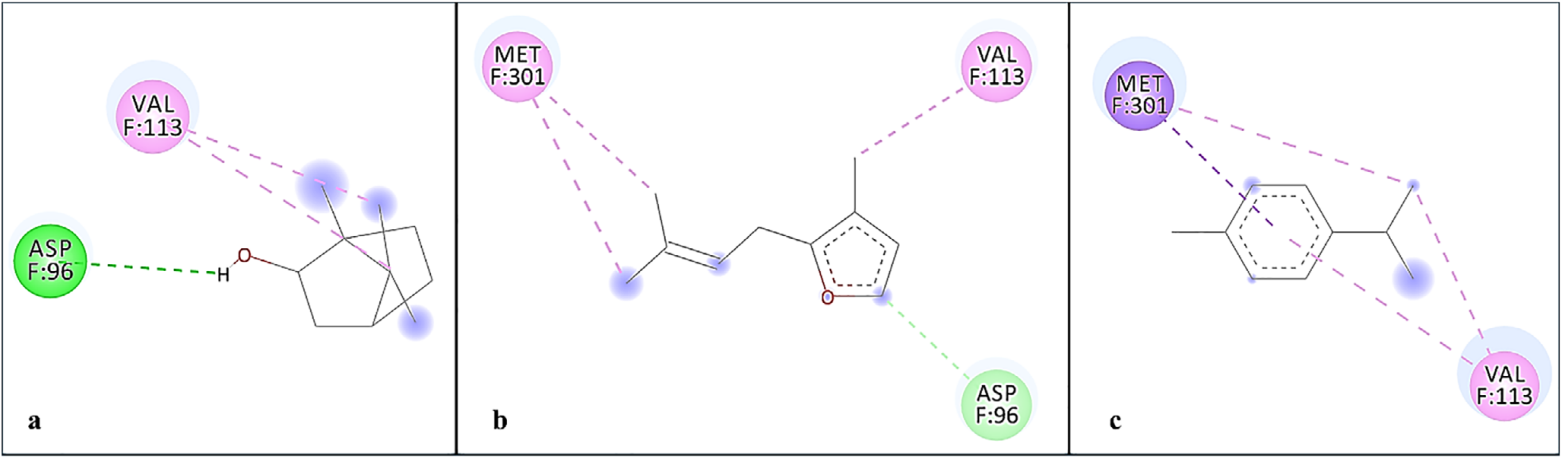
Interaction between ligands and DNA gyrase A (PDB ID:4DDQ) enzyme (a) Borneol, (b) *γ*‐Clausenane, (c) *p*‐Cymene.

For the 4DDQ (DNA gyrase A) receptor, docking energies were −4.8 kcal/mol for both Borneol and *γ*‐Clausenane and −5.0 kcal/mol for *p*‐Cymene. RCSB PDB lists TRS (Tris) and K+ as the crystallographic small‐molecule components of 4DDQ; TRS is a buffer/crystallization component rather than a pharmacological inhibitor and therefore should not be interpreted as a reference inhibitor in the docking comparison. The comparable docking energies of the three terpenoids indicate similar accommodation within the selected 4DDQ pocket. In Figure 5a, Borneol can support a polar interaction with ASP96 together with a hydrophobic contact involving VAL113. For *γ*‐Clausenane (Figure 5b), which is treated in the present GC–MS classification as a sesquiterpene hydrocarbon, the interaction should be described through hydrophobic contacts involving VAL113 and MET301 rather than through a conventional hydrogen bond. *p*‐Cymene (Figure 5c) likewise engages the pocket primarily through π/hydrophobic contacts involving VAL113 and MET301. These docking‐derived residues were selected as the contact set to be examined in the short‐timescale molecular‐dynamics assessment described below.

In contrast, relatively lower binding affinities were recorded for the 3KP3 (transcriptional regulator TcaR) and 1U1Z (FabZ) receptors, where the energies ranged from −3.8 to −4.7 kcal/mol. This observation likely reflects reduced complementarity between the small, nonpolar terpenoids and the hydrophilic nature of these protein pockets.

For the four receptors in which conventional redocking validation was applicable, the study compounds were compared with the corresponding redocked co‐crystallized ligands obtained under the same docking conditions. For 2JA1, the study compounds yielded scores of −5.6 to −5.8 kcal/mol, close to that of redocked TTP (−6.3 kcal/mol). For 3JWB, the study compounds showed more negative scores (−5.2 to −5.3 kcal/mol) than redocked NLE (−3.8 kcal/mol), whereas for 3KP3 and 6WAA they were slightly weaker than redocked AIC (−5.2 kcal/mol) and TNJ (−6.8 kcal/mol), respectively. These redocking results were used primarily to validate and refine the binding‐site definition and to provide an internal reference for interpreting the docking profiles of the selected terpenoids.

Molecular docking against a panel of bacterial enzymes (including MurB/1HSK, thymidine kinase/2JA1, and DNA gyrase A/4DDQ) demonstrated that all three terpenoids can be accommodated within structurally relevant pockets primarily through hydrophobic and π‐type interactions. *p*‐Cymene displayed the most favorable docking score toward MurB (−6.7 kcal/mol), stabilized by extensive π–alkyl and π–π contacts within the FAD‐associated region; *γ*‐Clausenane was supported mainly by nonpolar contacts, while Borneol additionally offered a hydroxyl group capable of polar interaction. These interaction modes are consistent with the predominantly hydrophobic character of the selected volatile constituents and provide the residue‐level basis for subsequent trajectory‐based contact analysis. These interaction modes parallel mechanistic patterns previously described for terpenoid–enzyme complexes, where hydrophobic, π–π stacking, and hydrogen‐bonding interactions collectively stabilize ligand binding and contribute to enzyme inhibition in bacterial systems [74, 75, 76]. Recent studies have further supported the use of molecular docking and integrated computational approaches to investigate the biological potential and molecular interactions of natural compounds [72, 73, 77].

### 2.7 Short Molecular Dynamics Assessment

The 1 ns trajectories were evaluated as a short‐timescale consistency check of the docking poses rather than as evidence of long‐term conformational stability. For all three 4DDQ complexes, the protein‐backbone and ligand RMSD values remained approximately 2 Å, indicating that no major displacement of the docked complexes occurred during the simulated interval. Borneol, which contains a hydroxyl group, retained the docking‐derived ASP96/VAL113 interaction pattern, including the direct ASP96 hydrogen bond, with an occupancy of 68%. In contrast, *γ*‐Clausenane and *p*‐Cymene are nonpolar hydrocarbon ligands; therefore, their persistent interactions with VAL113 and MET301 were quantified as hydrophobic or π/hydrophobic contacts rather than direct protein–ligand hydrogen bonds. The corresponding contact occupancies were 53% for *γ*‐Clausenane and 47% for *p*‐Cymene (Table 9). Collectively, these results support the short‐timescale persistence of the principal docking‐derived contacts while avoiding interpretation of the 1 ns trajectories as evidence of long‐term conformational stability.

**TABLE 9:** Persistence of docking‐derived contacts during the 1 ns molecular‐dynamics simulations.

Compound	Docking‐derived residue(s)	Interaction monitored in MD	1 ns occupancy (%)
Borneol	ASP96; VAL113	Direct H bond to ASP96; hydrophobic contact with VAL113	68
*γ*‐Clausenane	VAL113; MET301	Hydrophobic contact (not a direct H bond)	53
*p*‐Cymene	VAL113; MET301	π/hydrophobic contact (not a direct H bond)	47

## 3 Conclusion

This study characterized the chemical profile and biological activities of *O. minutiflorum* root essential oil and, for the first time, evaluated the antibacterial potential of its major volatiles using ADME, molecular docking, and short molecular dynamics approaches. The major constituents were *p*‐Cymene (36.7%), *γ*‐Clausenane (31.1%), and borneol (10.6%), providing a basis for future biological and industrial applications. These bioactive terpenoids suggest potential pharmacological and industrial uses of the essential oil, supporting further evaluation of its bioactivity and sustainable application. Furthermore, due to its antimicrobial and antioxidant activities, the essential oil of *O. minutiflorum* could serve as a natural preservative to extend the shelf life of food products. Overall, the computational data highlight that the principal components of OmEO possess suitable pharmacokinetic characteristics and favorable binding orientations within biologically relevant enzyme pockets. These findings suggest that *γ*‐Clausenane and *p*‐Cymene, in particular, could serve as lead scaffolds for the development of natural antibacterial agents. The short 1 ns molecular‐dynamics assessment further supported the persistence of the docking‐derived 4DDQ interaction patterns, with the highest monitored occupancy observed for Borneol (68%). The integration of ADME prediction, docking, and short‐timescale trajectory analysis thus provides a rational framework for prioritizing these plant‐derived volatile constituents for further experimental and longer‐timescale computational evaluation.

### 3.1 Experimental Section

#### 3.1.1 Chemicals, Plant Material, Extraction of Essential Oil

HPLC‐grade n‐hexane and anhydrous sodium sulfate were purchased from Sigma‐Aldrich (St. Louis, MO, USA) unless otherwise stated. A homologous series of *n*‐alkanes (C_6_–C_30_) was used for the calculation of retention indices under the same chromatographic conditions as the samples. All chemicals and solvents used in the study were of analytical or chromatographic grade.

The roots of *Origanum minutiflorum* O. Schwarz & P.H. Davis were collected from Diyarbakır. After collection, the roots were cleaned to remove soil and foreign materials, cut into small pieces, and used for essential oil isolation.

### 3.2 Hydrodistillation Procedure for the Isolation of Essential Oil

Approximately 80 g of fresh roots of *O. minutiflorum* were subjected to hydrodistillation using a modified Clevenger‐type apparatus for 3 h. The condenser system was maintained with a cooling bath at 7°C. After completion of the distillation, the obtained essential oil was separated from the aqueous phase and dried over anhydrous sodium sulfate. The essential oil yield was calculated as 0.625% (v/w) based on the fresh weight of the plant material. For GC‐FID/MS analysis, the oil was dissolved in HPLC‐grade n‐hexane and stored in a dark glass vial at 4°C–6°C until analysis [41].

### 3.3 Gas Chromatography‐FID/MS Analysis

The essential oil of *O. minutiflorum* (OmEO) was analyzed using a Shimadzu QP2010 Ultra GC‐FID/MS system, equipped with a PAL AOC‐5000 Plus autosampler and controlled by Shimadzu Class‐5000 Chromatography Workstation software. Separation was performed on a Restek Rxi‐5MS capillary column (30 m × 0.25 mm i.d., film thickness 0.25 µm, USA). The injection volume was 1 µL, and the sample was injected in split mode with a split ratio of 1:30. The injector temperature was set at 230°C. The oven temperature program was as follows: initial temperature of 60°C, held for 2 min, then increased to 240°C at 3°C/min, followed by a final hold at 250°C for 4 min. Helium was used as the carrier gas at a constant flow rate of 1 mL/min. Mass spectrometric detection was carried out in electron‐impact ionization mode at 70 eV, and mass spectra were recorded over the m/z 40–450 range. Retention indices (RIs) of the volatile constituents were calculated using the Kovats method based on a homologous series of n‐alkanes (C_6_–C_30_) under the same analytical conditions. Identification of the components was achieved by comparing their RI values with those in the literature and matching the mass spectra with reference libraries (NIST, Wiley 7NL, FFNSC 1.2, and W9N11) [31, 41, 42].

### 3.4 Determination of Total Phenolic Contents (TPC) by Folin–Ciocalteu Method

The TPC of the extract was measured using the method of Slinkard and Singleton, with minor modifications [78]. Catechin and gallic acid were used as standards, and results were expressed as µg catechin equivalent (CE) and gallic acid equivalent (GAE) per mL of sample.

### 3.5 Antioxidant Activity

#### 3.5.1 DPPH• Radical Scavenging Assay

The DPPH• radical scavenging assay was carried out with slight modifications following the method of Brand‐Williams, et al. [79]. Trolox and BHT were used as reference antioxidants for comparison. The results were expressed as IC_50_ (µg/mL), with lower values indicating more potent antioxidant activity.

#### 3.5.2 ABTS•^+^ Radical Scavenging Assay

The ABTS•^+^ radical scavenging capacity of the extract was determined spectrophotometrically at 734 nm, following the method of Re et al. [80]. The results were expressed as IC_50_ values (µg/mL), representing the sample concentration required to scavenge 50% of the ABTS•^+^ radicals.

#### 3.5.3 Ferric Reducing/Antioxidant Power (FRAP) Assay

The FRAP assay was performed according to the procedure of Benzie and Strain [81], using Trolox as a standard. Results were expressed as µM Trolox equivalent antioxidant capacity (TEAC).

#### 3.5.4 Antimicrobial Activity

The OmEO was diluted to 400, 300, 200, 100, 50, and 25 µg/mL by using DMSO (10%). The antibacterial activity of the essential oils at all doses was evaluated against four Gram‐positive [*Staphylococcus aureus* (ATCC 25923), *Bacillus cereus* (ATCC10876), *Bacillus subtilis* (ATCC6633), *Staphylococcus epidermidis* (ATCC12228)] and four Gram‐negative [*Klebsiella pneumoniae* (ATCC13883), *Citrobacter freundii* (ATCC43864), *Enterobacter aerogenes* (ATCC3048), *Pseudomonas aeruginosa* (ATCC9027)] supplied from the Department of Molecular Biology and Genetics/Erzurum Technical University. The disc diffusion and the microdilution methods were used to evaluate the antibacterial activity and the minimal inhibitory concentrations (MICs) described by [82]. OFX (Ofloxacin, 10 µg/disc) for the disc diffusion method and Maxipime (Bristol‐Myers Squibb) (3.90‐250 µg/mL) for the micro‐dilution method were used as the positive controls, and DMSO (10%) was used as the negative control for both. The sterile discs (6 mm) impregnated with 10 µL of the essential oils at all doses were placed on Tryptic Soy Agar (TSA) plates previously inoculated with 100 µL of a bacterial suspension containing approximately 10^8^ CFU/mL. The plates were then incubated at 37°C for 24 h. The antimicrobial activity of the essential oil was determined by measuring the diameter of the clear inhibition zones around the discs (in millimeters). All experiments were performed in triplicate.

#### 3.5.5 RAPD‐PCR Protocol

Wheat seeds were surface sterilized by soaking in 10% sodium hypochlorite for 10 min, followed by thorough rinsing with sterile distilled water. Uniform seeds were placed in sterile petri dishes lined with double layers of Whatman No. 1 filter paper, with 10 seeds per dish. Essential oils were applied at concentrations of 0.2, 0.4, 0.8, and 1.6 µL/mL, while the control group received sterile water containing Tween 20. After incubation at 25°C for one week, root and shoot lengths were measured. DNA was extracted following [83]. The RAPD protocol was adjusted according to previous studies [84]. From a total of tested 20 RAPD primers, 10 best binding primers were selected. Sequences of the primers are listed in Table 10.

**TABLE 10:** Sequences of primers used for RAPD analysis (5'–3').

1	5'–3'	GTGCTCGTGC
2	5'–3'	TCTCCGCCCT
3	5'–3'	CCTGGGTGGA
4	5'–3'	TTCCCCCGCT
5	5'–3'	CCCACATTCC
6	5'–3'	CAGGCCCTTC
7	5'–3'	AGGCAGAGCA
8	5'–3'	ACGGTACCAG
9	5'–3'	GGGCCAATGT
10	5'–3'	ACTTCGCCAC

PCR products were separated on 1% agarose gels containing ethidium bromide and visualized under UV light. RAPD profiles were analyzed using gel imaging software to assess band presence and intensity relative to negative controls. Genomic template stability (GTS) was calculated for all primer sets to evaluate genomic integrity.

### 3.6 Computational Details

#### 3.6.1 In Silico ADME Analysis

To assess the pharmacokinetic suitability of the major constituents identified in the OmEO, the absorption, distribution, metabolism, and excretion (ADME) characteristics of *p*‐Cymene, *γ*‐Clausenane, and Borneol were evaluated using the SwissADME online platform [85]. These compounds were selected based on their predominance in the essential oil and their known biological relevance in terpene‐based pharmacology. Canonical SMILES notations for each molecule were retrieved from the PubChem database and submitted to SwissADME for prediction. Key pharmacokinetic descriptors, including molecular weight, lipophilicity (logP), hydrogen bond donors and acceptors, topological polar surface area (TPSA), and gastrointestinal (GI) absorption, were calculated to estimate their drug‐likeness and bioavailability profiles. The three most abundant constituents—p‐Cymene (36.7%), γ‐Clausenane (31.1%), and Borneol (10.6%)—collectively accounted for 78.4% of the total essential‐oil composition; therefore, these three compounds were prioritized for the computational analyses.

### 3.7 Molecular Docking

The crystal structures of selected bacterial target proteins were retrieved from the Protein Data Bank [86]. The receptor dataset included enzymes that play key roles in essential cellular processes (Table 11), such as UDP‐N‐acetylenolpyruvylglucosamine reductase (MurB) (PDB ID: 1HSK [87]), (3R)‐hydroxyacyl‐acyl carrier protein dehydratase (FabZ) (PDB ID: 1U1Z [88]), glycerophosphodiesterase (PDB ID: 2DXN [89]), thymidine kinase (PDB ID: 2JA1 [90]), L‐methionine gamma‐lyase (PDB ID: 3JWB), transcriptional regulator TcaR (PDB ID: 3KP3 [91]), DNA gyrase A (PDB ID: 4DDQ [92]), and DNA topoisomerase IV (PDB ID: 6WAA [93]).

**TABLE 11:** Structural and biological details of the receptors used in docking studies.

PDB Code	X‐ray ligand	Organism	Receptor
1HSK	FAD (flavin‐adenine dinucleotide; cofactor)	*S. aureus*	UDP‐N‐acetylenolpyruvylglucosamine reductase (MurB)
1U1Z	SO4 (sulfate ion)	*P. aeruginosa*	(3R)‐hydroxyacyl‐acyl carrier protein dehydratase (FabZ)
2DXN	Zn2+ (zinc ion)	*E. aerogenes*	Glycerophosphodiesterase
2JA1	TTP (thymidine‐5'‐triphosphate)	*B. cereus*	Thymidine kinase
3JWB	NLE (norleucine)	*C. freundii*	L‐methionine gamma‐lyase
3KP3	AIC (ampicillin)	*S. epidermidis*	Transcriptional regulator TcaR
4DDQ	TRS (Tris)	*B. subtilis*	DNA gyrase A
6WAA	TNJ (compound 34)	*K. pneumoniae*	DNA Topoisomerase IV

All receptor structures were processed using AutoDock Tools [94] to prepare them for molecular docking. Non‐essential heteroatoms, crystallographic water molecules, and redundant co‐factors were removed, except for the native ligand, which was retained during the validation phase to define the active binding region. Hydrogen atoms were added to all polar residues, and Gasteiger partial charges were assigned. The processed receptor models were saved in PDBQT format for further computational analysis.

The three‐dimensional structures of *p*‐Cymene, *γ*‐Clausenane, and Borneol were retrieved from the PubChem database [95]. Initial hydrogenation and geometric optimization were conducted using Discovery Studio Visualizer [96] via the “Clean Geometry” protocol, which applies a Dreiding‐based force field for energy minimization.

Each ligand was further refined by assigning Gasteiger charges and defining all rotatable bonds as flexible to ensure accurate conformational sampling during docking. Finally, the optimized ligands were converted and stored in PDBQT format to ensure compatibility with AutoDock Vina and related docking algorithms.

Docking grid parameters were configured to encompass the native ligand‐binding sites, ensuring accurate coverage of the active pocket. Grid box sizes were defined according to the receptor dimensions listed in Table 12, with most systems adopting a grid spacing of 0.375 Å. The grid centers were aligned with the coordinates of the co‐crystallized ligands to preserve the biological relevance of each docking site.

**TABLE 12:** Grid box parameters for docking simulations.

PDB Code	Dimensions	x	y	z
1HSK	40x40x52	179.523	148.695	163.978
1U1Z	25x50x40	3.646	40.088	137.701
2DXN	50x50x40	32.998	38.328	7.741
2JA1	40x40x40	2.158	9.474	16.407
3JWB	40x40x40	11.6	18.303	11.879
3KP3	60x60x60	−39.86	5.958	9.657
4DDQ	40x40x40	22.763	−68.391	−50.976
6WAA	40x40x40	−36.394	−28.712	−155.316

### 3.8 Docking Protocol Validation

RCSB PDB inspection identified FAD in 1HSK, SO4 in 1U1Z, Zn2+ in 2DXN, TTP in 2JA1, NLE (norleucine) in 3JWB, AIC (ampicillin) in 3KP3, TRS (Tris) in 4DDQ, and TNJ (compound 34) in 6WAA as the crystallographic small‐molecule components. To revalidate the docking protocol, the biologically relevant co‐crystallized ligands TTP (2JA1), NLE (3JWB), AIC/ampicillin (3KP3), and TNJ/compound 34 (6WAA) were removed from their crystallographic complexes and re‐docked into the corresponding binding sites using the same AutoDock Vina workflow applied to the study compounds. The re‐docked co‐crystallized ligands yielded binding scores of −6.3, −3.8, −5.2, and −6.8 kcal/mol for 2JA1–TTP, 3JWB–NLE, 3KP3–AIC, and 6WAA–TNJ, respectively. The crystallographic ligand centroids and pocket extents were then used to refine the grid‐box centers and dimensions reported in Table 12. FAD in 1HSK is a bound cofactor, whereas SO4 (1U1Z), Zn2+ (2DXN), and TRS/K+ (4DDQ) are not appropriate drug‐like redocking benchmarks and were therefore used only as structural references for pocket definition.

Molecular docking analyses were carried out using AutoDock Vina [97] to predict the binding affinity and orientation of the ligands within the active sites of the selected receptors. The docking setup followed a protocol like previously reported methodologies [98] with specific grid box dimensions and coordinates listed in Table 12. For each receptor–ligand complex, a minimum of twenty binding conformations were generated, and the pose displaying the lowest binding free energy (kcal/mol) was selected as the most plausible interaction mode.

The resulting docking complexes were further examined using UCSF Chimera [99] and Discovery Studio Visualizer. The interaction profiles were analyzed to identify key molecular contacts, including hydrogen bonding, hydrophobic interactions, π–π stacking, and electrostatic attractions. The binding affinities obtained from Vina scoring were compared across all tested compounds to determine those with the most promising inhibitory potential against the target bacterial proteins.

### 3.9 Molecular Dynamics Simulations

Short all‐atom molecular‐dynamics simulations were performed for the three 4DDQ complexes obtained with Borneol, *γ‐*Clausenane, and *p*‐Cymene. The docked complexes were parameterized in the AMBER framework using ff19SB for the protein and GAFF2‐compatible ligand parameters with AM1‐BCC partial charges. Each complex was placed in a periodic truncated‐octahedral box of TIP3P water extending at least 10 Å from the solute and neutralized with counterions. After staged energy minimization, the systems were heated from 0 to 300 K under NVT conditions, equilibrated under NPT conditions at 300 K and 1 atm, and subjected to a 1 ns production run using a 2 fs integration step. Bonds involving hydrogen atoms were constrained with SHAKE, long‐range electrostatics were treated by particle‐mesh Ewald, and a 10 Å cutoff was applied to short‐range nonbonded interactions. Trajectory analysis focused on short‐timescale complex stability and persistence of the docking‐derived contacts. Direct hydrogen‐bond occupancy was evaluated using donor–acceptor distance ≤3.5 Å and donor–H–acceptor angle ≥135°, while nonpolar contacts were reported separately for *γ*‐Clausenane and *p*‐Cymene.

### 3.10 Statistical Analysis

All data were expressed as mean ± standard deviation (SD). Statistical analyses were performed using IBM SPSS Statistics 22 software. Differences among groups were evaluated by one‐way analysis of variance (ANOVA), followed by Duncan's multiple range test for multiple comparisons. A significance level of *p* < 0.05 was used. Values sharing the same letter were considered not significantly different.

## Author Contributions

D.E. designed the study; A.A.K., G.Y.O, S.B. and B.K. performed experiments; D.E. and A.A.K. analyzed data; D.E, A.A.K and B.K. wrote the manuscript; all authors reviewed and approved the final version.

## Conflicts of Interest

The authors declare no conflicts of interest.

## Data Availability

The data that support the findings of this study are available on request from the corresponding author. The data are not publicly available due to privacy or ethical restrictions.
